# Environmental NO_2_ Level is Associated with 2-Year Mortality in Patients Undergoing Peritoneal Dialysis

**DOI:** 10.1097/MD.0000000000000368

**Published:** 2015-01-09

**Authors:** Jui-Hsiang Lin, Tzung-Hai Yen, Cheng-Hao Weng, Wen-Hung Huang

**Affiliations:** From the Department of Nephrology and Division of Clinical Toxicology (J-HL, T-HY, C-HW, W-HH), Chang Gung Memorial Hospital, Linkou Medical Center; Chang Gung University College of Medicine (T-HY, C-HW, W-HH); and Division of Nephrology (J-HL), Department of Internal Medicine, Taoyuan General Hospital, Ministry of Health and Welfare, Taoyuan, Taiwan, R.O.C.

## Abstract

An ongoing issue related to global urbanization is the association of air pollution with increased incidences of morbidity and mortality. However, no in-depth study has investigated this issue focusing on peritoneal dialysis (PD) patients. Therefore, this study assessed the effects of traffic-related air pollutants and other important mortality-associated factors on 2-year mortality in PD patients.

A total of 160 PD patients were recruited in this 2-year retrospective observational study. Differences in air quality were analyzed with respect to the patients’ living areas. The PD patients were categorized into 2 groups according to high (n = 65) and low (n = 95) nitrogen dioxide (NO_2_) exposure. Demographic, hematological, nutritional, inflammatory, biochemical, air pollutants, and dialysis-related data were analyzed. Univariate and multivariate Cox regression analyses were used for 2-year mortality analysis.

A total of 160 PD patients (38 men and 122 women) were enrolled. Fourteen patients (8.8%) died within 2 years; among them, the causes of death were infection (n = 10), malignancy (n = 1), and cardiovascular events (n = 3). Among the 10 patients who died from infection, 5, 4, and 1 died from pneumonia, PD-related peritonitis, and sepsis of unknown origin, respectively. All patients who died from pneumonia were living in high environmental NO_2_ exposure areas. Multivariate Cox regression analysis showed that age (hazard ratio [HR] 1.073, 95% confidence interval [CI] [1.013–1.137]; *P* = 0.017), white blood cell count (HR 1.41, 95% CI [1.116–1.781]; *P* = 0.004), log normalized protein nitrogen appearance (HR 0.0001, 95% CI [0–0.073]; *P* = 0.005), high cardiothoracic ratio (HR 14.28, 95% CI [1.778–114.706]; *P* = 0.012), and high environmental NO_2_ exposure (HR 3.776, 95% CI [1.143–12.47]; *P* = 0.029) were significantly associated with 2-year mortality.

PD patients with high environmental NO_2_ exposure had a higher 2-year mortality rate than those with low exposure. Therefore, air pollution may be associated with 2-year mortality in such patients.

## INTRODUCTION

As a result of global urbanization, air pollution has become a global issue. Fossil-fueled emissions from motor vehicles have skyrocketed in recent years along with rapid urbanization. Thus, there is an ongoing issue regarding the effects of air pollution on human health. In particular, the increasing number of days in which air pollution levels exceed the level suggested by the World Health Organization's guidelines on air quality has recently attracted attention.^1^ Understanding the consequences of air pollutants on public health is essential for developing policies to decrease ambient air pollution. Previous studies described the effects of air pollution on daily excess deaths or mortality risk using time-series methods.^2,3^ Air pollution exposure can lead to various complications and diseases associated with increased risks of cerebrovascular incidents and specific cancers.^4,5^ In addition, air pollution is associated with increased incidences of morbidity and mortality due to cardiovascular system diseases.^6^ Furthermore, epidemiological evidence suggests that outdoor air pollution contributes to such morbidity and mortality.^7^ In the ongoing Netherlands Cohort Study on diet and cancer, Hoek et al^8^ reported that traffic-related air pollution is clearly related to cardiopulmonary mortality. In Canada, a growing body of epidemiologic evidence indicates that traffic-related air pollution negatively affects health and is a substantial public health concern.^9^ Nitrogen dioxide (NO_2_), sulfur dioxide (SO_2_), particulate matter with an aerodynamic diameter of ≤2.5 μm (PM_2.5_), and particulate matter with an aerodynamic diameter of ≤10 μm (PM_10_) are estimated indicators of traffic-related air pollution.^10^ Pulmonary systemic diseases may occur in the setting of high traffic-related air pollution and are associated with the induction of oxidative stress.^11^

In end-stage renal disease, functional dependence, impaired intellectual status, diabetes, low serum albumin level, peripheral vascular disease, and late referral for treatment are poor prognostic factors.^12^ Our recent study focusing on air pollution levels in elderly patients undergoing maintenance hemodialysis revealed an association between mortality and living environment.^13^ However, no in-depth study on the effects of traffic-related air pollution on peritoneal dialysis (PD) patients has been reported. Therefore, this study assessed the effects of traffic-related air pollutants and other important mortality-associated factors on the 2-year mortality rate of PD patients in Taiwan.

## MATERIALS AND METHODS

This retrospective observational study complied with the guidelines of the Declaration of Helsinki and was approved by the Medical Ethics Committee of Chang Gung Memorial Hospital, a tertiary referral center located in Northern Taiwan. In addition, all individual information was securely protected by delinking identifying information from the main dataset and was available to investigators only. Furthermore, all data were analyzed anonymously, and all patients’ records and information were anonymized and deidentified before analysis. Finally, all primary data were collected according to the Strengthening the Reporting of Observational Studies in Epidemiology guidelines.

### Study Population

We randomly and retrospectively recruited 160 patients who had received continuous ambulatory PD (CAPD) or automated PD (APD) for at least 4 months and had been regularly followed for 2 years at the PD center in Chang Gung Memorial Hospital. Recruitment started in October 2009, and follow-up ended in December 2011. PD supplies (ie, CAPD and APD solutions) were obtained from Baxter Healthcare SA (Singapore). Patients who had developed dialysis-related peritonitis or active infection within 3 months before the study were excluded. Patients with a smoking habit were also excluded. All clinical data were obtained from the patients’ medical records. The primary end point was nonaccidental mortality.

### Sample Collection

Plasma, dialysate, and urine concentrations of creatinine, serum albumin, and urea nitrogen were measured using routine laboratory methods during annual routine examinations of PD patients. Protein nitrogen appearance was normalized to body weight. High cholesterol level was defined as cholesterol level ≥200 mg/dL, and high triglyceride level was defined as triglyceride level ≥150 mg/dL. Residual renal function was calculated as follows: (renal normalized urea nitrogen clearance + renal normalized creatinine clearance)/2. Anuria was defined as 24-hour urine volume <50 cm^3^. Corrected calcium concentration was calculated using the following equation: corrected calcium = serum calcium (mg/dL) + [0.8 (4.0 − serum albumin (g/dL)]. Education level was categorized as low (ie, less than senior high school) and high (ie, senior high school or above). Air pollutant levels were also categorized as high or low level according to their respective median values.

### Air Quality Status and Analysis

Data from the Taiwan Air Quality Monitoring Network, which is operated by the Environmental Protection Administration, including the database and report on the air quality status in previous 1 year (12 months) were analyzed.^14^ The differences in air quality with respect to the patients’ living areas were recorded and analyzed. The contributions of outdoor air pollution to morbidity and mortality are associated with both short-term^15,16^ and long-term exposure.^17–19^ According to the above-cited studies, the 1-year average exposure concentration of air pollutants was considered for each subject. The reference items included previous 1-year average concentrations of traffic-related air pollutants including PM_10_, and PM_2.5_, SO_2_, and NO_2_.^10^ Air quality was classified as high or low environmental exposure to air pollutants on the basis of the median of the previous 1-year average concentration of each air pollutant. Air pollution levels were recorded by a network of 25 monitoring stations near or in the patients’ living areas in 29 districts in Taiwan. Air pollutant data were generally obtained from monitoring stations in the same district. If a patient lived between 2 monitoring stations, the air pollutant data of the nearest station were selected for analysis. If a patient lived in a district without a monitoring station, the air pollutant data would be referenced from the nearest station (<15 km). Terrain was also considered; the data of the nearest monitoring station on the same side of a mountain that a patient lived on were analyzed. The levels of air pollutants were checked every hour for 1 year. Therefore, the average of approximately 8760 data points at every monitoring station was calculated to determine the previous 1-year average levels of air pollutants.

### Statistical Analysis

The Kolmogorov–Smirnov test showed all variables to be normally distributed. A *P* value >0.05 was required to assume a normal distribution. Nonnormally and normally distributed data are expressed as median (interquartile range) and mean ± SD, respectively. Comparisons between groups were performed using the Mann–Whitney *U* test and Student *t* test. The χ^2^ test or Fisher exact test was used to analyze the associations among categorical variables. The data of intact parathyroid hormone (iPTH), normalized protein nitrogen appearance (nPNA), and high-sensitivity C-reactive protein (hs-CRP) levels were log-transformed for analysis. Univariate and multivariate Cox regression analyses were used for 2-year mortality analysis; variables with *P* < 0.1 in univariate analysis were entered into the multivariate analysis (forward model). The following factors were analyzed: PM_2.5_, PM_10_; SO_2_; NO_2_; age; sex; PD duration; serum creatinine, calcium, albumin, and phosphate levels; serum white blood cell count (WBC); nPNA; cardiothoracic ratio (CTR); anuria condition; iPTH, blood aluminum (Al) level, diabetes mellitus (DM), coronary artery disease (CAD), education level, and hypertension. All statistical analyses were performed using SPSS version 12.0 for Windows (SPSS Inc, Chicago, IL). The level of significance was set at *P* < 0.05. Missing data were approached with listwise deletion.

## RESULTS

A total of 160 PD patients (38 men and 122 women; mean age, 50.4 ± 10.5 years) who met the inclusion criteria were enrolled. The baseline clinical characteristics of the patients as well as the annual means of particulate matter, SO_2_, and NO_2_ measured in PD patients are shown in Table 1. Eighty-three patients had a high education level, 19 had a medical history of DM, 3 had CAD, 73 had hypertension, 1 had cancer, 16 had hepatitis B virus infection, and 6 had hepatitis C virus infection. Regarding NO_2_ exposure, 95 and 65 patients lived with low and high environmental NO_2_ exposure, respectively. Fourteen patients (8.8%) died within 2 years, including 10, 1, and 3 from infection, malignancy, and cardiovascular events, respectively; among the 10 patients who died from infection, 5, 4, and 1 were due to pneumonia, PD-related peritonitis, and sepsis of unknown origin, respectively. All patients who died from pneumonia were living in areas with high environmental NO_2_ exposure.

**TABLE 1:**
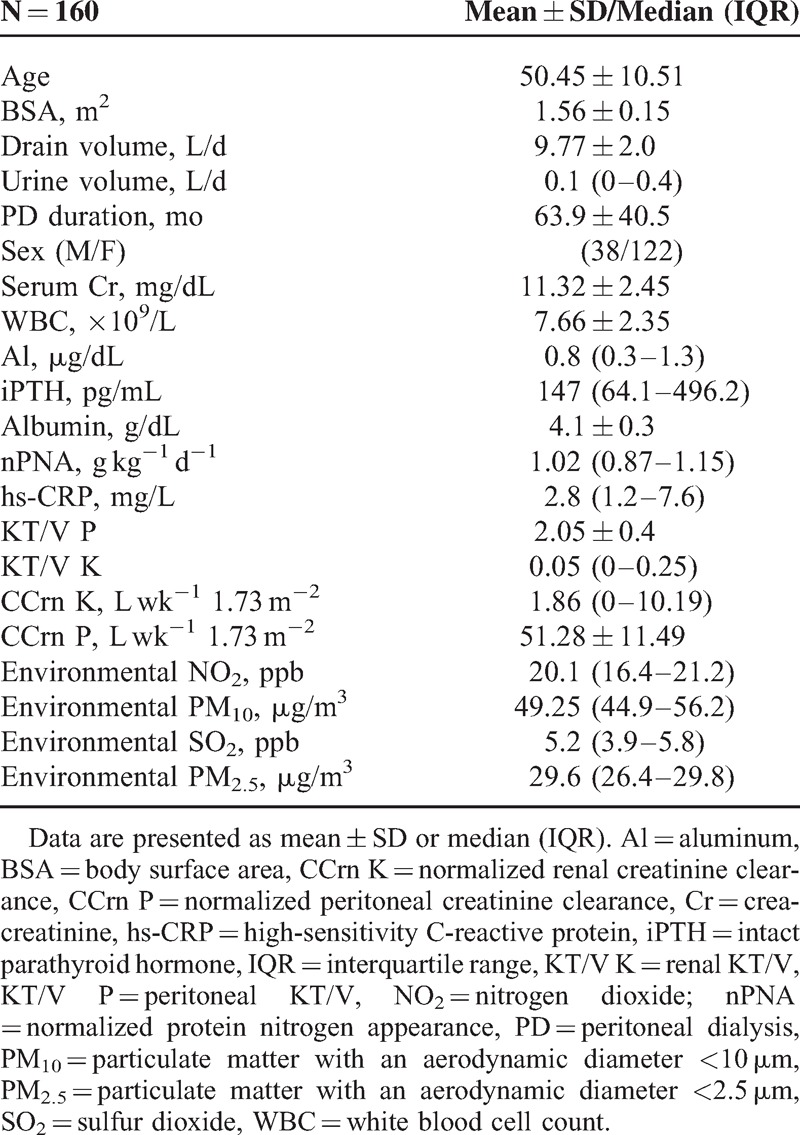
Patient Characteristics

Univariate Cox regression analysis was used to evaluate the association between mortality and clinical variables in the PD patients. As shown in Table 2, the initial univariate Cox regression analysis indicated that the following variables were significantly associated with 2-year mortality: age (hazard ratio [HR], 1.076; 95% confidence interval [CI], 1.026–1.127; *P* = 0.002), WBC (HR, 1.189; 95% CI, 1.01–1.4; *P* = 0.038), log nPNA (HR, 0.0001; 95% CI, 0–0.038, *P* = 0.002), high CTR (HR, 13.731; 95% CI, 1.796–104.982, *P* = 0.012), high education level (HR, 0.572; 95% CI, 0.355–0924, *P* = 0.022), DM (HR, 3.2; 95% CI, 1.003–10.208; *P* = 0.049), and high environmental NO_2_ exposure (HR, 3.833; 95% CI, 1.202–12.222; *P* = 0.023). Nevertheless, log hs-CRP levels (HR, 2.522; 95% CI, 0.99–6.422; *P* = 0.052), albumin (HR, 1.174; 95% CI, 0.23–6.001; *P* = 0.847), and comorbid diseases including CAD (HR, 1.217; 95% CI, 0.422–3.509; *P* = 0.716) and hypertension (HR, 2.215; 95% CI, 0.742–6.61; *P* = 0.154) were not significantly associated with 2-year mortality (Table 2). Variables with *P* < 0.1 in the univariate Cox regression were entered into the multivariate Cox regression analysis. The results show that the following variables were significant risk factors for 2-year mortality: age (HR 1.073, 95% CI [1.013–1.137]; *P* = 0.017), WBC (HR 1.41, 95% CI [1.116–1.781]; *P* = 0.004), log nPNA (HR 0.0001, 95% CI [0–0.073]; *P* = 0.005), high CTR (HR 14.28, 95% CI [1.778–114.706]; *P* = 0.012), and high environmental NO_2_ exposure (HR 3.776, 95% CI [1.143–12.47]; *P* = 0.029) (Table 2).

**TABLE 2:**
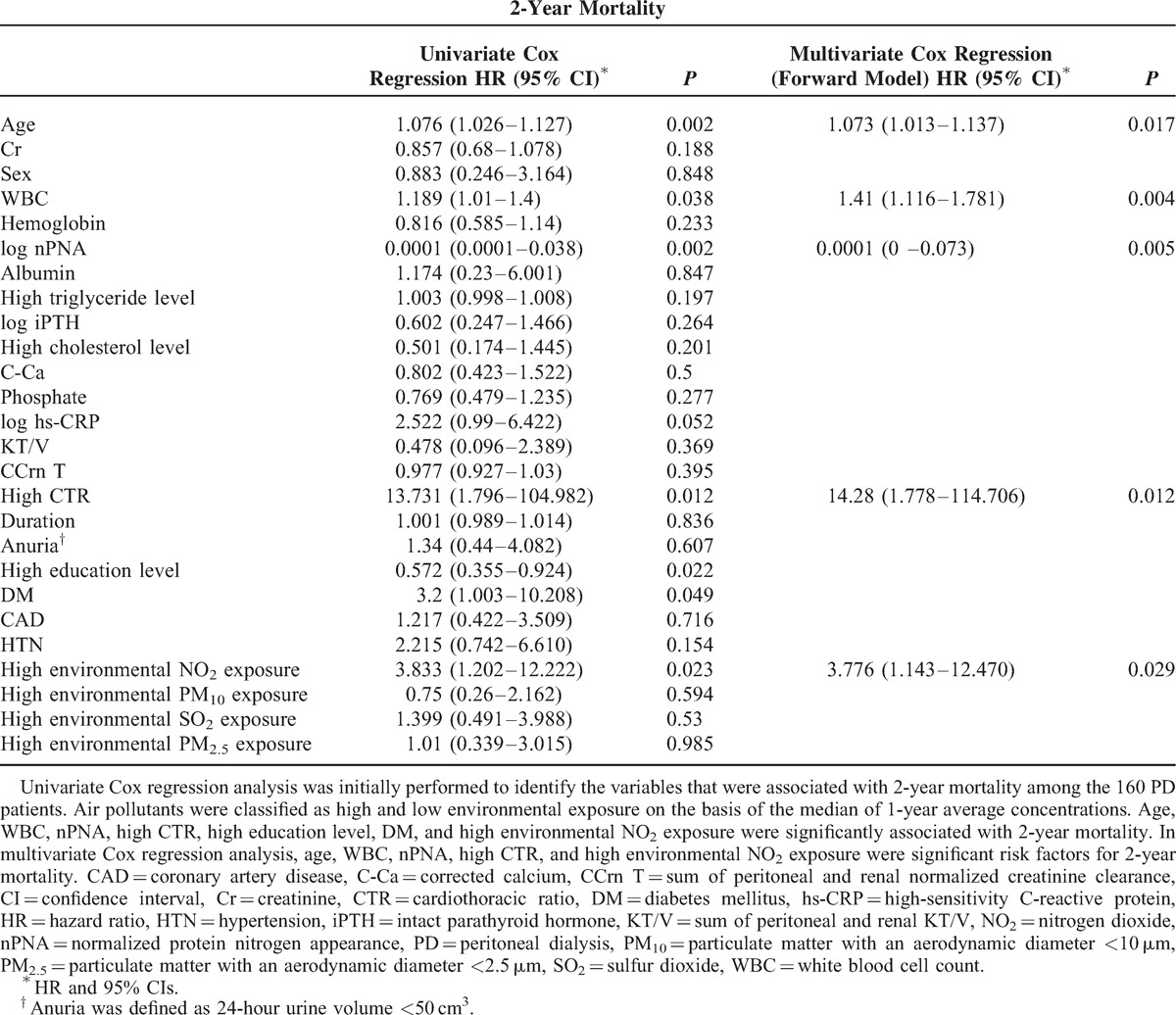
Cox Regression Analysis of the Associations of Variables With 2-Year Mortality (N = 160)

PD patients were divided into 2 subgroups according to the median NO_2_ concentration: low environmental NO_2_ exposure (n = 95) and high environmental NO_2_ exposure (n = 65). Age (49.66 ± 10.08 vs 51.6 ± 10.0 years), body surface area (1.56 ± 0.15 vs 1.57 ± 0.16 m^2^), PD duration (60.41 ± 38.54 vs 69.15 ± 43.02 months), WBC (7.71 ± 2.58 vs 7.59 ± 1.98  × 10^9^/L), serum albumin level (4.05 ± 0.34 vs 4.1 ± 0.29 g/dL), nPNA (1.02 ± 0.2 vs 1.03 ± 0.25 g kg^−1^ day^−1^), and hs-CRP levels (2.4 [1.12, 6.87] vs 3.42 [1.27, 9.38] mg/L) were not significantly different between patients living with low and high environmental NO_2_ exposure (Table 3).

**TABLE 3:**
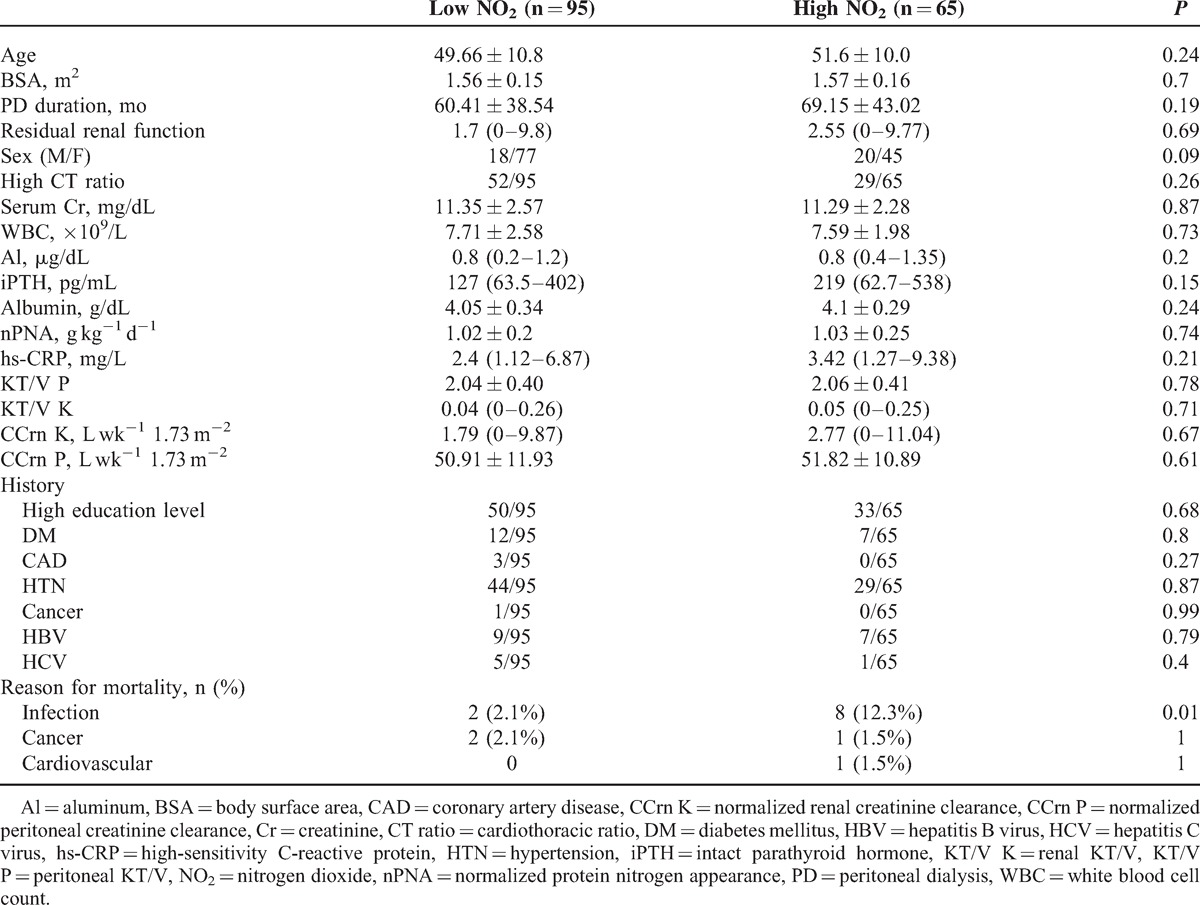
Comparison of Patients According to Environmental NO_2_ Exposure

**FIGURE 1:**
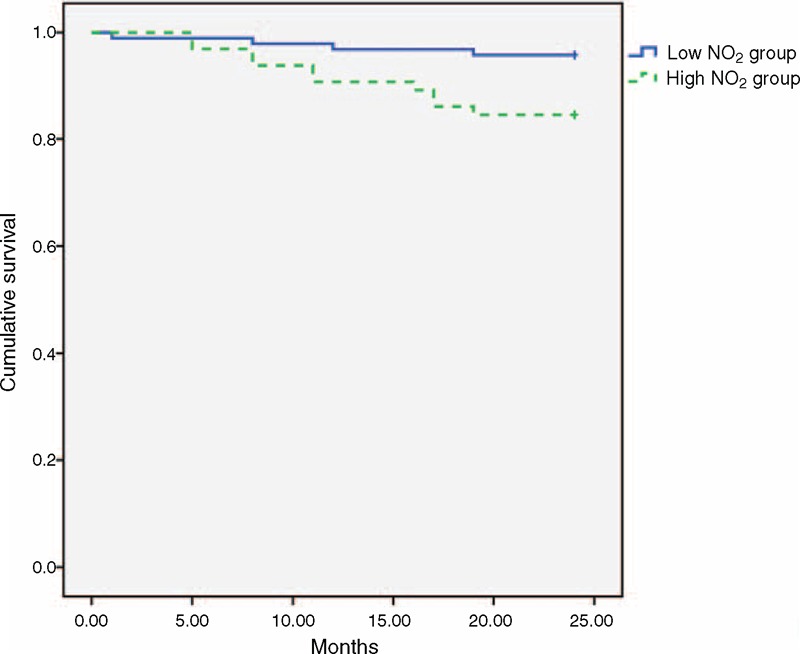
Kaplan–Meier survival analysis of patients living with high and low NO_2_ exposure levels. Patients living with high NO_2_ exposure levels suffered significantly higher cumulative mortality than those living with low NO_2_ exposure levels (log-rank test, χ^2^ test = 6.017; *P* = 0.014). NO_2_ = nitrogen dioxide.

Finally, Kaplan–Meier survival analysis showed that patients living with high NO_2_ exposure levels suffered significantly higher cumulative mortality than patients living with low NO_2_ exposure levels (log-rank test, χ^2^ test = 6.017; *P* = 0.014) (Figure 1).

## DISCUSSION

The present retrospective observational study indicates high environmental NO_2_ exposure is a significant predictor of 2-year mortality in nonsmoking patients undergoing PD after adjusting for age, leukocytes, nPNA, and high CTR. In their study on PD patients, Chen et al^20,21^ report that fasting blood sugar, age, nutrition status, and CTR are associated with 2-year mortality in patients undergoing maintenance PD. In the Dialysis Outcomes and Practice Patterns Study, 1-year mortality was associated with older age, catheter vascular access, albumin concentration, phosphorus concentration, cancer, and congestive heart failure.^22^ Therefore, age, nutrition status, and comorbidities are the most well-known predictors of mortality in dialysis patients.

With the effects of global urbanization, air pollution has become an unignorable public health issue. Interest in the effects of air pollution on health increased after 2 US cohort studies suggested that exposure to fine particulate matter in the air is associated with decreased life expectancy.^17,18^ In addition, mounting evidence indicates that exposure to air pollution may be associated with increased risks of adverse health effects. The study by Metzger et al^23^ published in 2004 indicates an association between exposure to poor-quality air and increased hospital admissions for cardiovascular diseases in the US. A series of the Harvard Six Cities study shows that cardiovascular and lung cancer mortality are positively associated with fine particulate air pollution concentrations (ie, PM_2.5_).^24^

Moreover, several studies have shown that traffic-related air pollution has substantial effects on public health.^8–10^ It is interesting to note that although traffic-related air pollution is associated with mortality in the general population, no study has discussed this issue on dialysis as an important variable for mortality. Our previous study regarding poor air quality in living environments showed that elderly hemodialysis patients living in a crowded metropolitan basin have elevated mortality rates.^13^ In the present study of 160 nonsmoking PD patients, PM_2.5_, PM_10_, NO_2_, and SO_2_ were used as traffic-related air pollutants. Univariate Cox regression analysis showed age, leukocyte count, DM, high CTR, nPNA, and high environmental NO_2_ exposure were significantly associated with 2-year mortality. Meanwhile, multivariate Cox regression analysis showed that 2-year mortality developed almost exclusively in PD patients with the abovementioned risk factors except DM. However, it should be mentioned that there was no evidence of an association between 2-year mortality in PD patients and comorbid illnesses such as DM, CAD, and hypertension in the present study.

The mechanisms by which air pollution contributes to mortality are not well understood. It is interesting to note that the patients living with high NO_2_ exposure had greater cumulative mortality than those living with low NO_2_ exposure. Literature on the association between mortality and environmental air quality in PD patients is limited. The densely populated urban areas generally have greater concentrations of air pollutants. The overdevelopment of urban areas, which entails increased traffic, production facilities, noise, density, and stress as well as poorer air quality, is not beneficial to human health. In Taiwan, there are an estimated 31,000 buses, 161,000 trucks, 6,091,000 passenger cars, 862,000 pick-up trucks, 61,000 specially constructed vehicles, and 15,139,000 motorbikes.^25^ In addition, air pollutants from neighboring China blow over Taiwan with the northeast monsoons. Therefore, the air quality of Taiwan must be addressed.

Over 3 million deaths per year are attributable to ambient air pollution worldwide; it is the 9th leading factor contributing to disease burden worldwide.^26^ Exposure to air pollutants such as NO_2_ can be considered an environmental risk factor for lung cancer and cardiopulmonary disease.^27,28^ Exposure to ambient air pollution is suggested to be associated with increased infection-related mortality, particularly pulmonary infections.^29^ Vieira et al^30^ report that exposure to higher levels of NO_2_ and O_3_ is associated with increased risks of asthma and pneumonia in children. In Vietnam, increased concentrations of NO_2_ and SO_2_ are associated with increased hospital admissions of young children for acute lower respiratory infections.^31^ In Japan, long-term exposure to NO_2_ was significantly associated with pneumonia in a study of 63,520 subjects.^29^ In older adults, exposure to ambient NO_2_ and PM_2.5_ is associated with hospitalization for community-acquired pneumonia.^32^ Accordingly, there is a positive association between NO_2_ exposure and pneumonia.

However, the mechanisms by which poor ambient air quality affects infections and immune systems are unclear. The relationship between immune events after infection onset and disordered immune responses led Blount et al^33^ to propose a unifying hypothesis relating respiratory infection to the immune system; they suggested that 2 air pollutants—PM_10_ and NO_2_—underlie suppressed Immunoglobulin M (IgM) responses. Meanwhile, Moreno et al^34^ showed that Immunoglobulin G (IgG), Immunoglobulin A (IgA) and Immunoglobulin M (IgM) are lower in urban residents than rural residents as a result of high concentrations of NO_2_ and PM_10_. However, in a case–control study of 52 subjects, subjects exposed to NO_2_ had elevated serum IgG levels as well as CD3^+^, CD4^+^, and CD8^+^ cells but decreased serum C3 and C4 levels.^35^ Schlesinger^36^ documented that the random mobility and phagocytic capacity of macrophages are decreased in rabbits exposed to NO_2_. However, the precise mechanisms underlying the acute inflammatory response to NO_2_ are not well understood either. It has been suggested that NO_2_, an oxidant pollutant, from air pollution exposure induces oxidative damage to cell membranes, resulting in the generation of reactive oxygen species and subsequent inflammation.^37,38^ These findings collectively demonstrate that increasing attention is being focused on the role of the immune system, providing evidence for the association between NO_2_ exposure and infection. This could explain why the patients living in high NO_2_ exposure areas had a higher infection-related mortality rate than those living in low NO_2_ exposure areas (12.3% vs 2.1%, *P* = 0.015). In addition, it is worth noting that among the 10 patients who died from infection in the present study, all 5 patients who died from pneumonia were in the high NO_2_ exposure group.

The present study has some limitations. First, the data were only from Taiwan; it is difficult to generalize the results to other countries. Second, it was difficult and complicated to compare survival with the mortality of PD patients, who have a higher prevalence of comorbidities (eg, infection, malignancy, and cardiovascular disease), in association with their living environments. Although some risk factors may not have been clarified yet, most well-known risk factors were included in this study. Third, the small sample size is a worth-noting limitation. In our PD center, there were around 400–450 patients in a cross-section period. All the subjects were selected at random in this study. The ratio of study patients is around 35.5% (160/450). These studied patients are representative of PD patients in our center. Fourth, in our PD center, choice of PD or HD was made by the patients themselves. In consideration of working environment and home care condition, most females chose PD. In the present study, most of the excluded smoking patients were men. In this study period, the total number of PD patients is 458 persons (F 280, M 178; the female:male ratio is 1.57:1). The sex ratio (F:M) of studied population is 3.2 to 1. It was not a coincidence. Fifth, according to the retrospective design in this study, assessment of how much and how often exposure occurred for a particular substance is difficult and a limitation of this study. However, in this study, there were 149 patients with CAPD and 11 patients with APD. We know that when patients receive CAPD, they need to change the peritoneal dialysate every 4 hours for 4 to 5 cycles. In this study, the mean drain volume of dialysate was about 9.77 L/d. It is very inconvenient for patients to carry such a large amount of liquid in the side. It means that the patients needed one place, which is always the self-house to exchange the dialysate. The range of activities of these patients is not usually too far from home. It is reasonable why air pollution levels were recorded by a network of the monitoring stations near or in the patients’ living areas in this study. Although our findings suggest that high NO_2_ exposures are related to an increased risk of mortality in PD patients, we cannot rule out the possibility of chance. Therefore, additional clinical trials are required to confirm the associations found in the present study.

## CONCLUSION

Patients undergoing PD and having high environmental NO_2_ exposure have a higher 2-year mortality rate than those with low environmental NO_2_ exposure. Therefore, air pollution may be associated with 2-year mortality in such patients.
